# Biochanin A Inhibits Glioblastoma Growth *via* Restricting Glycolysis and Mitochondrial Oxidative Phosphorylation

**DOI:** 10.3389/fonc.2021.652008

**Published:** 2021-07-08

**Authors:** Qiang Dong, Qiao Li, Lei Duan, Hang Yin, Xiaoqing Wang, Yang Liu, Bo Wang, Kun Li, Xuan Yao, Guoqiang Yuan, Yawen Pan

**Affiliations:** ^1^ Department of Neurosurgery, Lanzhou University Second Hospital, Lanzhou, China; ^2^ Key Laboratory of Neurology of Gansu Province, Lanzhou, China

**Keywords:** biochanin A, glioblastoma, energy metabolism, reactive oxygen species, proliferation

## Abstract

Abnormal metabolism serves a critical role in glioblastoma (GBM). Biochanin A (BCA), a flavonoid phenolic compound found in edible and herbal plants, has antioxidative and antitumor activities. However, it remains unclear whether BCA has an effect on energy metabolism. The aim of the present study was to evaluate the anticancer effects and molecular mechanism of the effect of BCA on energy metabolism. We observed that BCA inhibited the growth of U251 cells by the mitochondria-mediated intrinsic apoptotic pathway. BCA treatment reduced metabolic function, repressed mitochondrial membrane potential, and increased the production of reactive oxygen species (ROS) in GBM. In addition, we found that BCA decreased aerobic glycolysis by inactivation of the AKT/mTOR pathway. Taken together, the results demonstrate that treatment with BCA inhibited the proliferation of GBM by regulating metabolic reprogramming.

## Introduction

Glioblastoma multiforme (GBM) is the most common type of malignant primary brain tumor, with a median survival of only 14.6 months and 5-year survival of less than 5.5% (1). The standard treatment for GBM patients includes maximal safe neurosurgical resection and temozolomide (TMZ) chemotherapy with concomitant radiotherapy, followed by cycles of adjuvant TMZ (2). New non-toxic treatment strategies have become a research hotspot because the current treatment strategies are usually accompanied by serious side effects. Studies have shown that some small molecule drugs have therapeutic promise for a variety of cancers, including GBM through perturbation of cell death programs, lethal autophagy, metabolic reprogramming (3), and improvement of chemotherapy sensitivity (4). For example, plant-derived compounds ARTA and BETA displayed a significant cytotoxic impact on glioma cell migration (5); The 5,4’-dihydroxy-6,7,8,3’-tetramethoxyflavone compound (AB2) inhibits the growth of lung cancer cells by attenuation of mitochondrial membrane potential and activation of caspase-3 activity (4). However, the molecular mechanism of the occurrence and development of GBM is still poorly understood, and there are great advantages to address malignant disease phenotypes through the use of small molecule drugs.

Reactive oxygen species (ROS), a type of cellular metabolite, have important roles in biochemical functions (6). Excessive ROS promote DNA damage and trigger mitochondrial apoptosis (7). Due to the rapid growth of malignant tumors, there is not enough nutrition to satisfy tumor cells. Mitochondria produce ATP as energy required for conducting physiological processes, which can improve energy for tumor cells by dynamically regulating the fusion and division of mitochondrial morphology (8). Mitochondrial division and fusion are regulated by mitochondrial fusion proteins (MfN1, Mfn2, and OPA1) and mitochondrial division proteins (DRP1 and FIS1) (9). Glycolysis is a common feature of tumor cell metabolism (10). Even if there is an adequate oxygen supply, the malignant tumor also needs to get more energy *via* the glycolysis pathway, which is a phenomenon known as the “Warburg effect” (11). Meanwhile, mitochondria are involved in the regulation of metabolism and cell death and play an important role in tumor progression (12). Metabolic reprogramming of cancer cells plays an important role in maintaining the growth and proliferation of tumor cells (13–15). Recently, the metabolism of cancer cells has been considered a therapeutic hotspot for dietary and pharmacological interventions. The development of anti-tumor drugs with glycolysis inhibition and mitochondrial injury has important clinical significance for the prevention and treatment of glioma.

Biochanin A (BCA) is a methoxy isoflavone, which derives from the germinated germ part of chickpea, the heartwood of twining rosewood, single-leaf red bean, whole red clover, underground clover seedlings, soybean, alfalfa, peanut, and other legumes (16). BCA possesses a variety of biological activities, including antifibrotic (17), antioxidation (18), anti-inflammation (19), neuroprotection (20), the prevention of articular cartilage degeneration (21), and anticancer effects.

Increasing evidence suggested that natural products played a promising role in the development of novel chemotherapeutics for the treatment of cancers (22, 23). The previous study has reported the anti-proliferative effect of BCA by regulating various molecular mechanisms, such as the induction of apoptosis, cell cycle arrest, and suppression of ERK/AKT signaling (24, 25). BCA selectively sensitized cancer cells to apoptosis through inhibited cyclin D1 and arrested the cell cycle in G0/G1 phase (24). Moreover, BCA also regulated migration and invasion by suppressing the VEGF/VEGFR2 signaling pathway (26).

In this study, we evaluated the anticancer effects and molecular mechanisms of BCA in GBM. Considering the important role of energy metabolism in GBM cells, we further explored the mitochondrial oxidative phosphorylation and glycolysis in GBM cells. Additionally, we established the effect of BCA on intracellular ROS and mitochondrial division in GBM cells. Furthermore, we investigated the BCA anti-GBM activity in subcutaneous neoplasia in nude mice.

## Materials and Methods

### Reagents

BCA was purchased from Selleck Chemicals (Shanghai, China). Cell Counting Kit-8 (CCK-8) was obtained from Dojindo Molecular Technologies (Kumamoto, Japan). PE Annexin V apoptosis detection commercial kit was purchased from BD biosciences (Shanghai, China). Reactive Oxygen Species (ROS) Assay Kit was purchased from Beyotime Biotechnology (Shanghai, China). Cell-Light EdU Apollo567 In Vitro Kit was purchased from Guangzhou Ruibo Biotechnology Co. LTD (Guangzhou, China). Bax, Bcl-2, ND1, SHDB, UQCRC2, MTCO2, ATP5A, MFN2, GLUT1, HK2, PMK2, LDH, HIF-1a, and Drp1 antibodies were obtained from proteintech (Wuhan, China). AKT, p-AKT, mTOR, and p-mTOR antibodies were obtained from Cell Signaling Technology. LC3B, Beclin-1, P62, and GAPDH were obtained from Abcam (Cambridge, UK).

### Cell Culture

U251 cells were cultured in DMEM complete medium with high glucose and placed in an incubator at 37°C with 5% CO2. When the cell density is about 70–80%, a drug intervention is carried out.

### Cell Proliferation

U251 cells were inoculated in 96-well plates and treated with BCA for 48h and 72 h. Each well is added 10 μl of CCK8, incubated for 2 hours at 37°C incubator, and then detected absorbance with a microplate reader. U251 cells were seeded in 96-well plates, each well is added 100 μl of 50 uM Edu solution, incubated for 2 hours at 37°C incubator, and then 4% Paraformaldehyde fixation. After washing with PBS three times, each well is added to a 100 μl 1X Hoechst33342 solution, incubated at 37°C in the dark for 30 min, and is then observed and analyzed under a fluorescence microscope.

### Apoptosis Analysis

The percentage of apoptotic cells was tested by Annexin V-PE/FITC (BD, Biosciences). U251 cells were treated with 0, 50, and 100 μmol/L BCA for 48 h. Then, U251 cells (1 × 10^6^) were collected, after which 5 µl of PE Annexin V and 5 µl of 7-AAD were added. The cells were gently vortexed at room temperature and incubated for 15 minutes in the dark and the suspension was analyzed by flow cytometry (BD FACSCanto™ low cytometry, USA).

### Wound Healing Assay

BCA-treated U251 cells were inoculated into a 6-well plate. When the cells reached a confluence of 70–80%, cells were gently and slowly scratched with a new 200 ml pipette tip. The relative distance of the cells migrating was monitored and measured using a bright-field microscope at 0, 12, and 24 h. The experiments were repeated three times.

### Transwell Assay

Transwell chambers membrane was pre-coated with diluted Matrigel (1:8 BD biosciences). About 1×10^6^ cells in 100 μl serum-free medium were added into the top chambers, and 600μl of DMEM with 10% FBS was added to the lower transwell compartment. The cells on the chambers were fixed with 4% paraformaldehyde and stained with 0.1% crystal violet. Photos of the cells were taken using a bright-field microscope. Cell invasion assay was performed as above except used the cell culture inserts coated with Matrigel (BD Biosciences).

### Western Blot

U251 was harvested after being treated with drugs. After centrifuged, total protein was extracted with RIPA buffer, and concentration was examined *via* BCA protein analysis kit (Solarbio, PC0020). Then the samples were separated by SDS-PAGE, and transferred onto polyvinylidene fluoride (PVDF) membranes. The membranes were incubated with primary antibodies overnight at 4°C, and incubated with appropriate peroxidase-conjugated secondary antibodies for 1.5 h at room temperature, and then visualized by enhanced chemiluminescence with imageQuant LAS 500 system.

### Measurement of Oxygen Consumption Rate (OCR) and Extracellular Acidification Rate (ECAR)

For the oxygen consumption rate (OCR) measurement, U251 cells were seeded in the seahorse cell plate 20000/well and incubated overnight. Before an examination, the media were changed into 500 μl assay media (pH 7.4), which consisted of 10 mM glucose, 1 mM pyruvate, and 2 mM glutamine in XF Base Medium. Inhibitors of electron transport chain (ETC) complexes were added into different ports of the seahorse cartridge, including Oligomycin A (oligo, 1 μM), Carbonyl cyanide-4-(trifluoromethoxy)phenylhydrazone (FCCP, 1 μM), antimycin A (AA, 0.5 μM), rotenone (rot, 0.5 mM), then the OCR value was measured with the XF24 Seahorse Biosciences Extracellular Flux Analyzer (Seahorse Bioscience, 102238-100). Five replicates were repeated in each experimental group for analysis.

### ROS Measurements

To evaluate intracellular ROS level, U251 cells were incubated using 10 μM DCFH-DA (Solarbio, CA1410) for 20 min at 37°C after BCA treatment, then washed with serum-free medium three times and imaged under Olympus fluorescence microscope (BX53). Meanwhile, the cell was digested with trypsin and resuspended. The ROS level was analyzed by flow cytometry (BD FACSCanto™ low cytometry, USA).

### Mitochondrial Morphology

To observe mitochondrial morphology, the U251 cells were crawled and treated with BCA for 48 hours, stained with 10 nM MitoTracker at 37°C for 30min, fixed with 4% paraformaldehyde, and then observed and analyzed under a fluorescence microscope.

### Transmission Electron Microscopy

U251 cells were treated with BCA for 48 hours. Cells collected by trypsinization were fixed with 2.5% glutaraldehyde, followed by 1%OsO4. After dehydration, thin sections were stained with uranyl acetate and observed under a transmission electron microscope (JEM-1230, JEOL, Japan).

### Animals Experiments

Male BALB/c nude mice aged 4 weeks were purchased from Beijing Charles River and bred under SPF conditions. A total of 1×10^6^ U251 cells were dissolved in 0.1 mL medium to make cell suspension, and each nude mouse was injected into the right middle and posterior axilla. BALA/c nude mice were randomly divided into two groups (five mice per group), the control group (PBS 100 µL) and the BCA group (BCA 50mg/kg 100 µL). When the size of the subcutaneous tumor is 5×5 mm, the drug is intraperitoneally administered once a day for 2 weeks. The tumors were weighed, and volumes were counted using the equation V= (ab^2^)/2 (a: the longest axis (mm), b: the shortest axis (mm)).

### Histology and Immunohistochemistry Analysis

At 30 days after tumor inoculation, all animals were sacrificed, and their subcutaneous tumors were excised, fixed in 4% paraformaldehyde, and embedded in paraffin. Sections that were 5 μm thick were stained with hematoxylin & eosin (HE) and immunohistochemical staining.

### Statistical Analysis

The data were analyzed using SPSS22.0 software. All the figures were performed using GraphPad Prism software. The Student’s t-test and One-way Analyses of Variance (ANOVA) with a Tukey’s post-hoc test were used to assess group differences. Error bars represent the standard error of the mean (SEM). A P value < 0.05 was considered to be statistically significant.

## Results

### BCA Inhibited GBM Cells Growth, Migration, and Invasion

**Figure 1:**
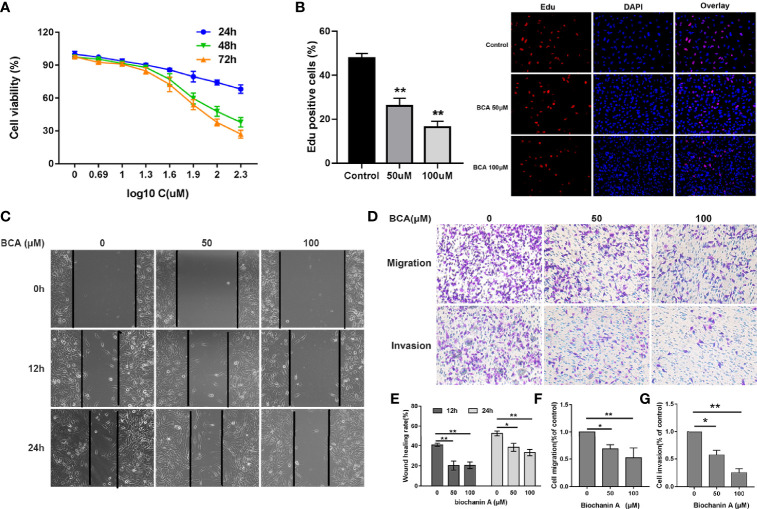
BCA inhibited the proliferation, migration, and invasion of U251 cells. **(A)** U251 cells were treated with various concentrations of BCA for 24, 48, and 72 h. Cell proliferation was measured by CCK8 assay. **(B)** Cellular proliferation was measured *via an* Edu assay. **(C)** Wound healing assay shows the migrated cells at 0, 12, and 24 h after treatment with BCA (0, 50, and 100 μM). **(D)** After treatment, the transwell assay showed that the migration and invasion cells at 24 h. **(E)** Quantification of the wound healing rate in A after treatment with BCA. **(F, G)** Quantification of the migration and invasion cells. (∗) p < 0.05 and (∗∗) p < 0.01 for Student’s t test.

To evaluate the cytotoxic effect of BCA, U251 cells were seed in 96-well plates and treated with different concentrations of BCA for 24, 48, and 72 h. Cell viability decreased in a concentration-dependent manner after treatment with BCA by CCK8 assay (**Figure 1A**). In addition, the Edu assay was performed to determine the effect of BCA on glioma cell proliferation. BCA treatment significantly increased the percentage of Edu-positive cells compared with the control (**Figure 1B**). Taken together, these data indicated that BCA inhibited the growth of U251 in a concentration-dependent manner. Furthermore, we further explored whether BCA has an effect on cell invasion and migration in U251 cells. U251 were cultured and then co-incubated with different doses (0, 50, and 100 μM) of BCA for various time intervals (0, 12, and 24 h). Wound healing assay showed that after treatment of BCA for 12 and 24 h significantly decreased cell migration rates in U251 cells (**Figures 1C, E**). We also examined cell migration and invasion capacity using a transwell chambers system after the indicated cell lines were treated with different doses (0, 50, and 100 μM) of BCA (**Figures 1D, F, G**). Migration and invasion rates of U251 significantly decreased after BCA treatment, which is consistent with the cell wound healing assay. These results showed that BCA inhibits U251 migration and invasion *in vitro*.

### BCA Increased ROS Generation and Decreased Mitochondrial Membrane Potential

**Figure 2:**
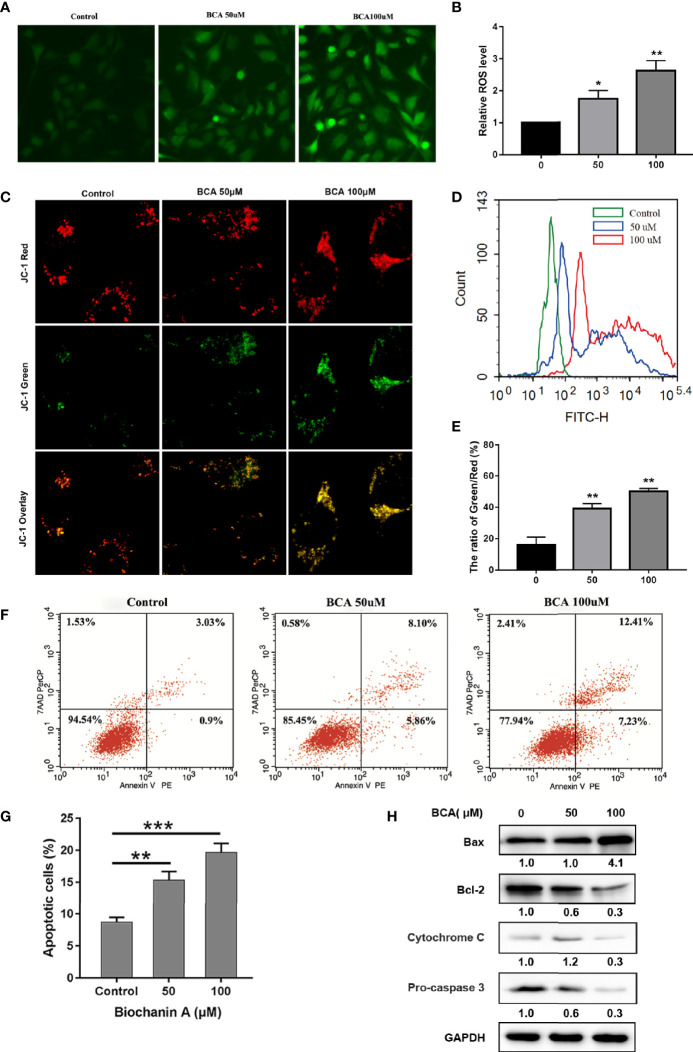
BCA induces apoptosis of U251 by increasing ROS levels and decreasing the mitochondrial membrane potential. **(A)** U251 was incubated with 0, 50 and 100 μM BCA for 48 h, The ROS level were observed under a fluorescence microscope after DCF-DA staining; **(B)** Quantification of relative fluorescence intensity in A; **(C)** U251 cells were incubated with 0, 50 and 100 μM BCA for 48 h, mitochondrial membrane potential was observed after JC-1 staining. **(D)** U251 cells were incubated with 0, 50 and 100 μM BCA for 48 h, then subjected to flow cytometric analysis of ROS levels after DCF-DA staining; **(F)** Quantification of the green and red fluorescence intensity rate in C. **(F)** The level of cell apoptosis was detected by flow cytometry. **(G)** The percentage of cell apoptosis ratio in A. **(H)** Expression levels of apoptotic related proteins (Bax, Bcl-2, cytochrome c, pro-caspase 3) at different concentrations of BCA. Data are expressed as mean ± SD. *P < 0.05, **P < 0.01, ***p < 0.001. *versus* control.

As oxidative stress plays an important role in inducing apoptosis of tumor cells, we then verified whether the ROS levels were related to BCA treatment in U251. Compared with control cells, the ROS level increased significantly in BCA treatment groups (**Figures 2A, B, D**). In the mitochondrial oxidative respiratory chain, the complex can pump hydrogen ions from the mitochondrial matrix into the mitochondrial space, thus forming an electric potential difference between the mitochondrial membrane space and the mitochondrial matrix. The complex V can use the electric potential to help itself to synthesize ATP. Therefore, the mitochondrial potential difference indirectly reflects the mitochondrial ability to synthesize ATP. The green fluorescence increases significantly after treatment of BCA, which means the mitochondrial potential was inhibited (**Figures 2C, E**). Taken together, these data indicated that BCA treatment triggers mitochondrial dysfunction in U251.

### Mitochondrial Apoptosis Is Activated by BCA in Human U251

Some small molecule compounds play antitumor effects mainly by inducing cell apoptosis. To verify whether BCA inhibited proliferation in an apoptosis-related manner. Annexin V and PE double staining assay was used to identify the apoptosis of the U251 cells after BCA treatment 48 h. BCA application increased the percentages of apoptosis, compared to the control group (**Figures 2F, G**). To further detect molecular markers related to apoptosis, expression of apoptosis-related proteins was determined in U251 cells following BCA treatment. Application of BCA induces the expression of Bax and a decrease in the level of cytochrome c, pro-caspase 3, and Bcl-2 (**Figure 2H**). Thus, these findings show that cytotoxic effects of BCA on U251 cells were partly caused by activation of the mitochondria-mediated intrinsic apoptotic pathway.

### BCA Increased Mitochondrial Fission and Decreased Mitochondrial Oxidative Phosphorylation

**Figure 3:**
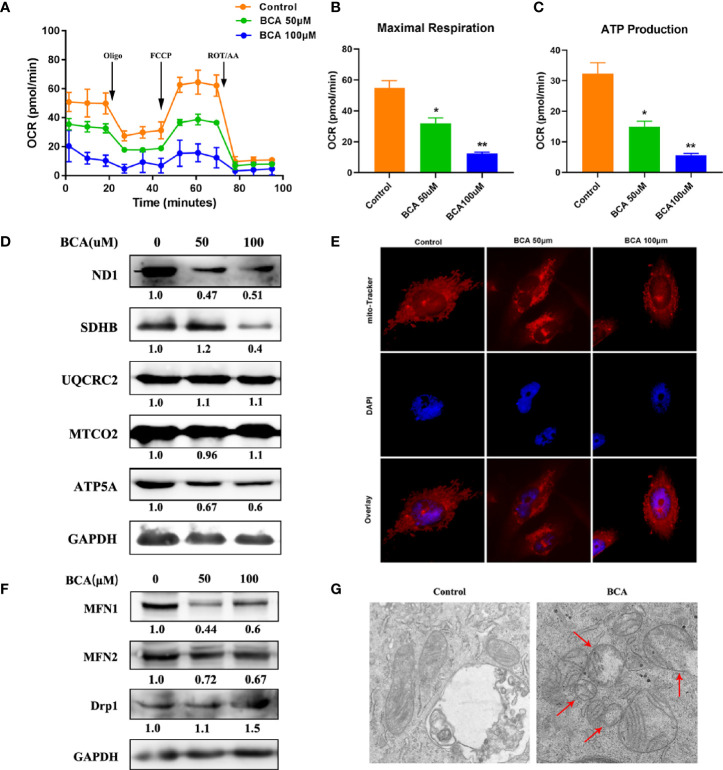
BCA increases mitochondrial fission and decreases mitochondrial oxidative phosphorylation. **(A)** Mitochondrial stress test to detect mitochondrial energy metabolism and respiratory functions in BCA (0, 50, 100 uM) group; **(B)** Quantification of the mitochondrial maximal respiration in A; **(C)** Quantification of the mitochondrial ATP production in A; **(D)** Western blot analysis the relative proteins of Mitochondrial respiratory chain (ND1, SDHB, UQCRC2, MTCO_2,_ ATP5A, and GAPDH). **(E)** Mitochondria morphology was observed by mito-Tracker staining; **(F)** Western blot analysis of the relative proteins of mitochondrial fusion division (MFN1, MFN2, and Drp1); **(G)** The morphology of mitochondria was observed by transmission electron microscope in control and BCA group. *P < 0.05, **P < 0.01 versus control.

The morphology of mitochondria plays an important role in regulating cell metabolism. So, we examined whether BCA intervention affects mitochondrial morphology and function by using MitoTracker staining. The number of mitochondria is large and filamentous morphology with tight cristae in the control group (**Figure 3E**). However, after BCA treatment, the mitochondria became smaller, punctate, and significantly shortened in length. Meanwhile, the electron microscope results showed that the mitochondrial morphology became smaller and vacuolated after BCA treatment (**Figure 3G**). To gain insight into the mechanism by which BCA regulates mitochondrial dynamics, we examined the expression of mitochondrial dynamics-related proteins, including MFN1, MFN2, and Drp1. After treatment of BCA, the western blotting results show that mitochondrial fusion protein MFN1 and MFN2 expression significantly decrease and division protein Drp1 expression significantly increase (**Figure 3F**). Fragmented mitochondria cause the dysfunction of mitochondrial metabolism. Next, we assessed mitochondrial function in cells using a Seahorse Extracellular Flux XF24 Analyzer. We observed in the oxygen consumption rate curves, both the basal and maximal mitochondrial respiratory capacities decreased in the BCA treatment group compared with the control group (**Figures 3A–C**). ATP production was also reduced in the BCA treatment group. Furthermore, we found that BCA treatment reduced the expression of ND1, SDHB, and ATP5A (**Figure 3D**). Together, these results showed that BCA might promote intracellular ROS and mitochondrial division and restrain oxidative phosphorylation of mitochondria in U251 cells.

### BCA Decreased the Glycolytic Capacity of U251 Cells

**Figure 4:**
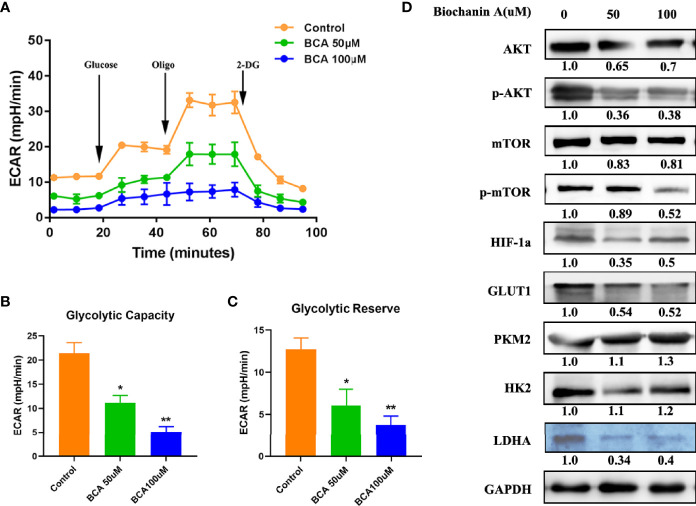
BCA decreases the Glycolytic Capacity of U251 cells. **(A)** Glycolytic stress test to detect glycolytic activities in BCA (0, 50, 100 uM) group; **(B)** Quantification of the glycolytic capacity in A; **(C)** Quantification of the glycolytic reserve in A; **(D)** Western blot analysis of relative proteins of Glycolytic (AKT, p-AKT, mTOR, p-mTOR, HIF-1a, GLUT1, PKM2, HK2, and LDHA). *P < 0.05, **P < 0.01 versus control.

The Warburg effect, characterized by abnormal metabolic phenomena that enhance glycolysis and reduces oxidative phosphorylation, induces significant differences between cancer cells and normal cells and affects tumor progression (27). Thus, after treatment of BCA, the capacity of glycolysis of U251 cells was examined using Seahorse XF24 extracellular flux analyzer. The capacity of glycolysis was significantly decreased in BCA treatment cells (**Figures 4A–C**). Some studies have shown that the AKT/mTOR/HIF-1α pathway played a vital role in glycolysis (14, 28, 29). A previous study has demonstrated that BCA can inhibit the activity of the PI3K/AKT signaling in U251cells (30). Our results also showed that the phosphorylation of both AKT and mTOR as well as the expression of HIF-1α were significantly decreased in U251 cells with BCA treatment. By testing relative protein glucose metabolism pathways, we found that BCA treatment reduced the expression of Glut-1, HK2, and LDHA (**Figure 4D**). Together, these results clearly indicated that BCA might decrease glycolysis inU251 cells by inhibiting the Akt/mTOR/HIF-1α signaling pathway.

### BCA Can Suppress Tumor Growth in Nude Mice

**Figure 5:**
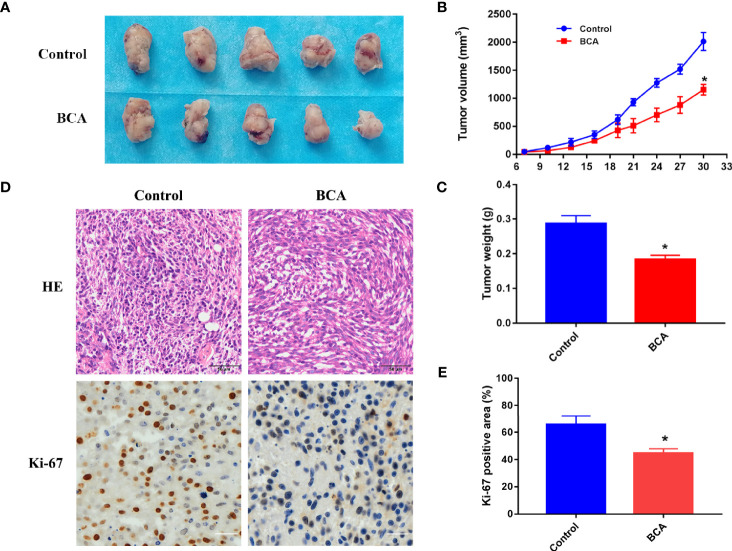
BCA inhibits tumor growth in nude mice. **(A)** The tumor of control and BCA treatment group. **(B)** Tumor volumes of the control and BCA treatment group were measured and calculated every 3 days. **(C)** The tumor weight was measured in the control and BCA treatment groups. *P < 0.05, **P < 0.01 compared with control group. **(D)** H&E stained tumor xenograft tissues in control and BCA treatment and immunohistochemistry was used to detect the expression of Ki-67 in tumor xenograft tissues. **(E)**. Quantification of Ki-67 positive rate in the different treatment groups. *p < 0.05 vs. control group.

The xenograft nude mouse model of U251 was established, which was used to evaluate the anti-tumor effect of BCA *in vivo*. We found that the tumor volume and quality of the BCA group were markedly inhibited by intraperitoneal injection of BCA (**Figures 5A–C**). Immunohistochemical (IHC) staining was performed to detect the expression of Ki67. The expression of Ki67 was significantly decreased in the BCA treatment group (**Figures 5D, E**). Meanwhile, H&E staining (**Figure 5D**) showed looser tumor tissue of mice with BCA treatment. Taken together, our results suggested that BCA inhibited tumor growth *in vivo*.

## Discussion

The Bcl-2 protein family contains two subclasses of proteins: one is: apoptosis-inhibiting proteins (such as Bcl-2, Bcl-Xl, and Bcl-Xy); the other is apoptosis-promoting proteins (such as Bax, Bix, and Bad, etc.). This present study demonstrated that BCA treatment increased the level of Bax and decreased the expression of Bcl-2, resulting in apoptosis induction. The activation bcl-2/bax ratio, cytochrome C release, and Cleaved-Caspase 3 is involved in the mitochondria-mediated intrinsic pathway in apoptosis (31). Flow cytometry experiments confirmed that the apoptosis rate of glioma cells increased markedly after BCA treatment. We further verified that the BCA inhibited the migration and invasion in U251cells by wound healing and transwell chamber assays.

The Warburg effect theory believes that even in the case of sufficient oxygen, tumor cells usually exhibit energy metabolism based on glycolysis (11). Metabolic changes caused by mitochondrial dysfunction, hypoxia, and carcinogenic signals make malignant tumor cells have better proliferation activity and production capacity in microenvironments such as hypoxia (27). In addition, the acidic tumor microenvironment associated with lactic acid accumulation due to increased glycolysis provides a tissue environment for the selection of cancer cells with high viability and malignant behavior (32). These changes in tumor biology and microenvironment pose great challenges for cancer treatment. The glucose uptake capacity of many tumor tissues is higher than that of neighboring normal tissues (33). Therefore, regulating tumor cell glycolysis and inhibiting the mitochondrial respiratory chain has become an important way to fight tumors. Blocking energy metabolism pathways may affect cell cycle activity, thus inhibiting cell proliferation, and promoting its apoptosis (34, 35). In order to further verify the relationship between energy metabolism and cell proliferation and apoptosis, the effect of BCA on energy metabolism of U251 cells was investigated by detecting glycolysis rate and mitochondrial pressure. The results showed that BCA could inhibit the glycolysis rate and the potential respiration capacity of mitochondria in U251 cells.

Previous research had reported that mitochondrial fusion enhanced oxidative metabolism, ATP production, and down-regulated ROS. The mitochondrial division increases glucose uptake and ROS level and weakens oxidative phosphorylation after exposure to chemotherapy and/or radiation therapy (36, 37). Besides, with the oxidative damage of cancer cells, an insufficient energy supply, intracellular calcium overload, and activation of apoptosis signals often lead to mitochondrial damage (38). Promotion of mitochondrial fission will be possible as a key method to prevent cancer progression. Consistent with previous research, our study found that the application of BCA triggered division, which also inhibited the survival rate of U251 cells *in vitro*.

We observed that mitochondrial ATP production and membrane potential decreased, ROS production increased, triggering mitochondrial apoptosis. Thereby, from a therapeutic perspective, BCA can active the mitochondrial division and is critical for anticancer drug development. AKT is a serine/threonine kinase that phosphorylates (activation or inactivation) downstream targets and plays an important role in cancer growth and metabolism (39). Some studies have shown that Akt activity is associated with the promotion of the Warburg effect. The PI3K/AKT/mTOR signaling pathway has been shown to be associated with the upregulation of HIF-1α (40). It can upregulate the transcription of glucose transporters and almost all glycolytic enzymes, such as hexokinase 2 (HK2), Pyruvate kinase isozyme type M2 (PKM2), and lactate deoxygenase (LDH) (41). The present study shows that BCA treatment obviously inhibits the expression of p-Akt and p-mTOR in glioma cells by Western blot detecting. Meanwhile, downstream molecules of the HIF-1a level of Glut-1, HK2, and LDHA were significantly decreased. These findings indicated that BCA-induced reduced Akt activity plays a vital role in inhibiting the levels of some glycolytic enzymes (Glut-1, HK2, and LDHA) and leads to reduced aerobic glycolysis in glioma cells.

**Figure 6:**
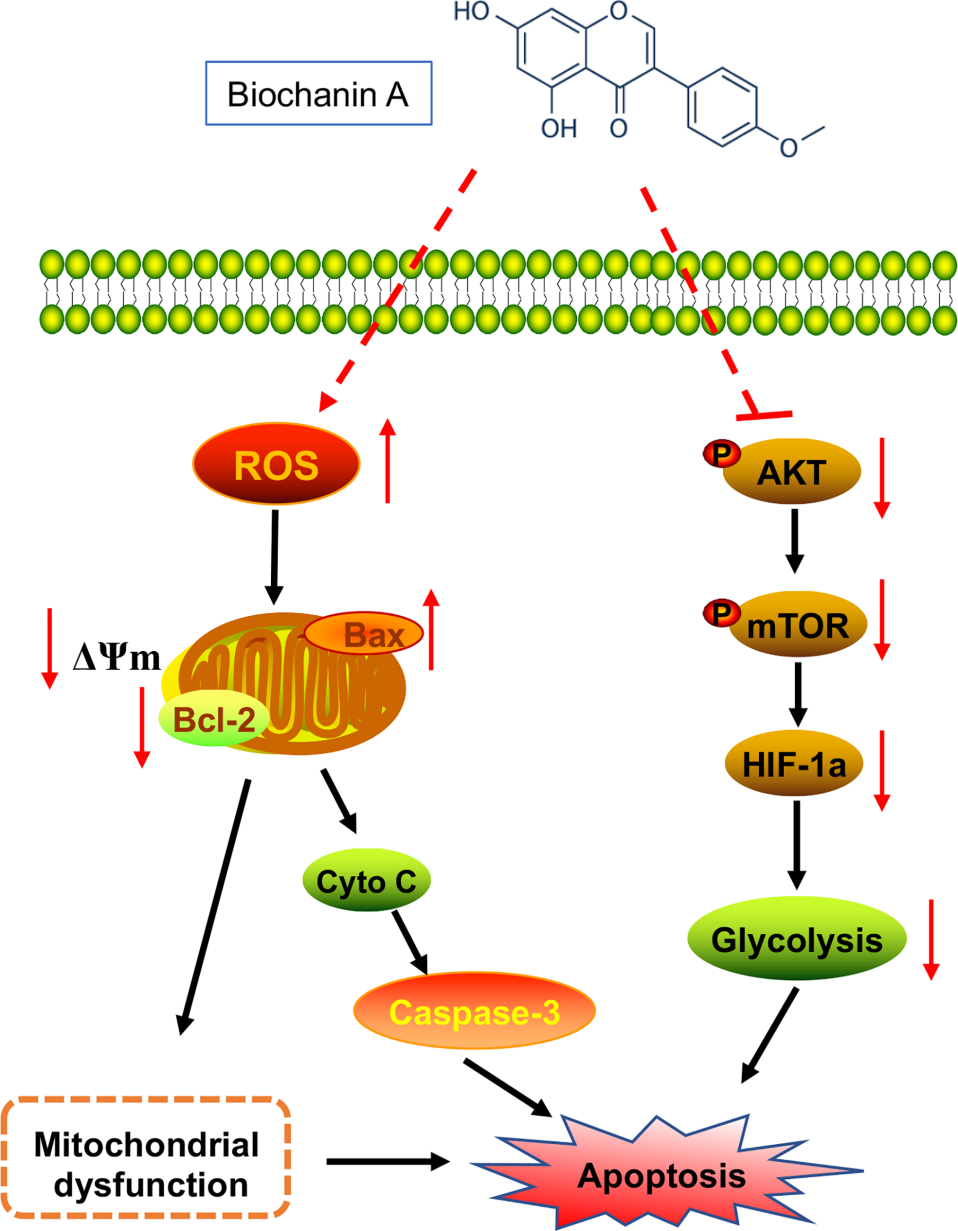
Schematic of the proposed mechanism of BCA inhibits glioblastoma proliferation by inducing metabolic reprogramming.

In conclusion, we have demonstrated in this study the strong anti-tumor activity of BCA both *in vitro* and *in vivo* by increasing intracellular ROS and mitochondrial division and inhibiting aerobic glycolysis in glioma cells. In addition, BCA treatment significantly inhibits the Warburg effect in U251 human glioma cells by regulating HIF-1a expression through the inactivation of the AKT/mTOR pathway (**Figure 6**). Taken together, these findings suggest that BCA may provide significant benefit in the treatment of glioma by metabolic reprogramming.

## Data Availability Statement

The original contributions presented in the study are included in the article/supplementary material. Further inquiries can be directed to the corresponding authors.

## Ethics Statement

The animal study was reviewed and approved by The Medical Ethics Committee of The Affiliated Second Hospital of Lanzhou University.

## Author Contributions

YP, GY, and QD conceived the project. QD, QL, LD, HY, BW, and KL performed the experiments. QD, QL, XW, YL, and XY analyzed the data. QL, GY, QL, XW, and LD interpreted the data and revised the manuscript. YP, GY, QL, and QD wrote the manuscript. All authors read and approved the final manuscript.

## Funding

This work was supported by grants from the National Natural Science Foundation of China (81960541/82060455), the Natural Science Foundation of Gansu Province (18JR3RA309/18JR3RA365/20JR10RA741/20JR10RA766), the Science and Technology Research Project of Gansu Province (145RTSA012 and 17JR5RA307), the Project of Healty and Famliy Planing Commission of Gansu (GSWSKY-2014-31/GSWSKY-2015-58/GSWSKY2018-01), the Lanzhou Science and Technology Bureau Project (2018-1-109), the Cuiying Science and Technology fund (CY2017-MS12/CY2017-MS15/CY2017-BJ15/CYXZ-01), Cuiying Graduate Supervisor Applicant Training Program (201803) and Special fund project for doctoral training (YJS-BD-13) of Lanzhou University Second Hospital.

## Conflict of Interest

The authors declare that the research was conducted in the absence of any commercial or financial relationships that could be construed as a potential conflict of interest.
